# Supplementary material for: Cross-sectional analysis of use of real-world data in single technology appraisals of oncological medicine by the National Institute for Health and Care Excellence in 2011–2021
Source: BMJ Open. 2024 Mar 14;14(3):e077297. doi: 10.1136/bmjopen-2023-077297 (PMC10941141; doi:10.1136/bmjopen-2023-077297)
Supplement: Supplementary data [file bmjopen-2023-077297supp001.pdf]

Supplement 1. Components in use of real-world data in data extraction

Type of use	Elements
Non-parametric use	Characteristics of population
	Treatment sequence
	Choice of comparators
	Health state
	Model cycle
	Survival distribution (intervention)
	Survival distribution (comparators)
	Time-to-discontinuation (intervention)
	Time-to-discontinuation (comparators)
Parametric use	Overall survival (OS) of intervention
	Progression-free survival (PFS) of intervention
	Response rate (intervention)
	Time-to-progress (intervention)
	Adverse event (intervention)
	Overall survival (OS) of comparators
	Progression-free survival (PFS) of comparators
	Response rate (Comparators)
	Time-to-progress (Comparators)
	Adverse event (Comparators)
	Transition probability
	Health utility (generic measure)
	Health utility (cancer specific measure)
	Disutility
	Resource use of health state cost
	End-of-life resource use
	Resource use of adverse event cost (intervention)
	Volume of treatment (intervention)
	Dose adjustment (intervention)
	Resource use of adverse event cost (comparators)
	Volume of treatment (comparators)
	Dose adjustment (Comparators)

Kang J, Cairns J. *BMJ Open* 2024; 14:e077297. doi: 10.1136/bmjopen-2023-077297

Supplement 2

Table 2A. Description of patterns of non-parametric use of real-world data

Pattern	Number (%)
No use of RWD	136 (59.39%)
Validating survival distribution of intervention and comparators	20 (8.73%)
Choice of comparators	14 (6.11%)
Validating survival distribution of comparators	13 (5.68%)
Treatment sequence	7 (3.06%)
Characteristics of population	7 (3.06%)
Validating survival distribution of intervention	4 (1.75%)
Treatment sequence & validating survival distribution of intervention and comparators	4 (1.75%)
Choice of comparator & validating survival distribution of intervention and comparators	3 (1.31%)
Choice of comparator & validating survival distribution of comparators & time-to-discontinuation of comparators	2 (0.87%)
Treatment sequence & time-to-discontinuation of intervention and comparators	2 (0.87%)
Treatment sequence & validating survival distribution of comparators	2 (0.87%)
Other*	15 (6.55%)
Total	229 (100%)

Kang J, Cairns J. *BMJ Open* 2024; 14:e077297. doi: 10.1136/bmjopen-2023-077297

Supplement 2

Table 2B. Description of patterns of parametric use of real-world data

Patterns	Number (%)
No use of RWD	55 (24.02%)
Estimating end-of-life cost	23 (10.04%)
Estimating OS of intervention and comparators	17 (7.42%)
Using RWD for estimating end-of-life & health state cost	14 (6.11%)
Using RWD for estimating health state cost	13 (5.68%)
Estimating OS & PFS of intervention and comparators	10 (4.37%)
Estimating end-of-life cost & dose adjustment of intervention and comparators	9 (3.93%)
Using RWD for estimating OS of intervention and comparators & end-of-life & health state cost	6 (2.62%)
Estimating OS & PFS of intervention and comparators & health state cost	6 (2.62%)
Estimating volume of treatment of intervention and comparators	4 (1.75%)
Estimating OS & PFS of comparators	3 (1.31%)
Estimating OS of intervention and comparators & end-of-life cost	3 (1.31%)
Estimating OS of intervention and comparators & health-state cost	3 (1.31%)
Estimating OS & PFS of intervention and comparators & health state cost & end-of-life cost	3 (1.31%)
Other*	60 (26.2%)
Total	229 (100%)

Kang J, Cairns J. BMJ Open 2024; 14:e077297. doi: 10.1136/bmjopen-2023-077297
