# Supplementary material for: Cross-sectional analysis of use of real-world data in single technology appraisals of oncological medicine by the National Institute for Health and Care Excellence in 2011–2021
Source: BMJ Open. 2024 Mar 14;14(3):e077297. doi: 10.1136/bmjopen-2023-077297 (PMC10941141; doi:10.1136/bmjopen-2023-077297)
Supplement: Author's manuscript [file bmjopen-2023-077297.draft_revisions.pdf]

BMJ Open

BMJ Open is committed to open peer review. As part of this commitment we make the peer review history of every article we publish publicly available.

When an article is published we post the peer reviewers' comments and the authors' responses online. We also post the versions of the paper that were used during peer review. These are the versions that the peer review comments apply to.

The versions of the paper that follow are the versions that were submitted during the peer review process. They are not the versions of record or the final published versions. They should not be cited or distributed as the published version of this manuscript.

BMJ Open is an open access journal and the full, final, typeset and author-corrected version of record of the manuscript is available on our site with no access controls, subscription charges or pay-per-view fees (http://bmjopen.bmj.com).

If you have any questions on BMJ Open's open peer review process please email info.bmjopen@bmj.com

BMJ Open

A review of use of real-world data in single technology appraisals of oncologic medicine by the National Institute for Health and Care Excellence in 2011 – 2021

Journal:	*BMJ Open*
Manuscript ID	bmjopen-2023-077297
Article Type:	Original research
Date Submitted by the Author:	30-Jun-2023
Complete List of Authors:	Kang, Jiyeon; London School of Hygiene and Tropical Medicine Faculty of Public Health and Policy, Health Services Research and Policy<br>Cairns, John; London School of Hygiene and Tropical Medicine, Department of Health Services Research and Policy
Keywords:	Health economics < HEALTH SERVICES ADMINISTRATION & MANAGEMENT, Health policy < HEALTH SERVICES ADMINISTRATION & MANAGEMENT, Decision Making, ONCOLOGY

SCHOLARONE™
Manuscripts

I, the Submitting Author has the right to grant and does grant on behalf of all authors of the Work (as defined in the below author licence), an exclusive licence and/or a non-exclusive licence for contributions from authors who are: i) UK Crown employees; ii) where BMJ has agreed a CC-BY licence shall apply, and/or iii) in accordance with the terms applicable for US Federal Government officers or employees acting as part of their official duties; on a worldwide, perpetual, irrevocable, royalty-free basis to BMJ Publishing Group Ltd ("BMJ") its licensees and where the relevant Journal is co-owned by BMJ to the co-owners of the Journal, to publish the Work in this journal and any other BMJ products and to exploit all rights, as set out in our licence.

The Submitting Author accepts and understands that any supply made under these terms is made by BMJ to the Submitting Author unless you are acting as an employee on behalf of your employer or a postgraduate student of an affiliated institution which is paying any applicable article publishing charge ("APC") for Open Access articles. Where the Submitting Author wishes to make the Work available on an Open Access basis (and intends to pay the relevant APC), the terms of reuse of such Open Access shall be governed by a Creative Commons licence – details of these licences and which Creative Commons licence will apply to this Work are set out in our licence referred to above.

Other than as permitted in any relevant BMJ Author's Self Archiving Policies, I confirm this Work has not been accepted for publication elsewhere, is not being considered for publication elsewhere and does not duplicate material already published. I confirm all authors consent to publication of this Work and authorise the granting of this licence.

For peer review only

A review of use of real-world data in single technology appraisals of oncologic medicine by the National Institute for Health and Care Excellence in 2011 – 2021

Jiyeon Kang[1], John Cairns[1]

[1]Department of Health Service Research and Policy,

Faculty of Public Health and Policy,

London School of Hygiene and Tropical Medicine, London, UK

Corresponding author: Jiyeon Kang (Jiyeon.Kang@lshtm.ac.uk)

15-17 Tavistock Place, London, WC1H 9SH, UK

Word count: 4,091

Keywords: Real-world data (RWD), Health Technology Assessment (HTA), single technology appraisal (STA), economic evaluation, oncology medicine, National Institute for Health and Care Excellence (NICE)

Abstract

Objectives This study aims to identify how real-world data (RWD) have been used in single-technology appraisals (STAs) by the National Institute for Health and Care Excellence (NICE).

For peer review only

Design Cross-sectional study of NICE technology appraisals of cancer drugs for which guidance was issued between January 2011 and December 2021 (n=229). The appraisals were reviewed following a published protocol to extract the data about the use of RWD. The use of RWD was analysed by reviewing the specific ways in which RWD were used and by identifying different patterns of use.

Primary outcome measure The number of appraisals where RWD are used in the economic modelling.

Results Most appraisals used RWD in their economic models. The parametric use of RWD was commonly made in the economic models (76% of the included appraisals), whereas non-parametric use was less common (41%). Despite widespread use of RWD, there was no dominant pattern of use. Three sources of RWD (registries, administrative data, chart reviews) were found across the three important parts of the economic model (choice of comparators, overall survival and volume of treatment).

Conclusions NICE has had a long-standing interest in the use of RWD in STAs. A systematic review of oncology appraisals suggests that RWD have been widely used in diverse parts of the economic models. Between 2011 and 2021, parametric use was more commonly found in economic models than non-parametric use. Nonetheless, there was no clear pattern in the way these data were used. As each appraisal involves a different decision problem and the ability of RWD to provide the information required for the economic modelling varies, appraisals will continue to differ with respect to their use of RWD.

For peer review only - http://bmjopen.bmj.com/site/about/guidelines.xhtml

Strengths and limitations of this study

· This study is the first to review how real-world data (RWD) have been used in economic models of oncology medicines appraised by the National Institute for Health and Care Excellence based on 11 years of evidence.

· This study systematically applied a protocol specially developed for data extraction.

· While the findings are likely to be less relevant to other disease areas, some insights with respect to the opportunities to use RWD and its limitations are transferrable.

Introduction

Real-world data (RWD) are increasingly being used in the life cycle of drug development (1). Clinical trials mainly generate the evidence to support decision-making in regulatory and health technology assessment (HTA), whereas RWD have been used complementarily for post-marketing surveillance. Interest in adopting and implementing RWD in the context of HTA has exploded in recent years (2) as the evidence gaps when relying heavily on clinical trials (3,4) are more widely recognised. Many HTA bodies view RWD as acceptable sources of data in the context of HTA (5,6). In most European HTA organisations, RWD are accepted to inform epidemiological data, cost, and resource use (7) and are used to fill evidence gaps by supporting the assumptions in economic models (8). Health economics and outcome research organisations, such as the European Network for HTA (EUnetHTA) and the International Society for the Professional Society for Health Economics and Outcomes Research (ISPOR), have been active in proposing how RWD can be collected and analysed in the HTA context (9–12).

For peer review only - http://bmjopen.bmj.com/site/about/guidelines.xhtml

The National Institute for Health and Care Excellence (NICE) has also shown increasing interest in RWD (13). NICE has highlighted the value of RWD and wants to use RWD to improve understanding of health care, resolve gaps in knowledge and drive early access to innovations (14). In June 2022, NICE introduced a real-world evidence (RWE) framework for the more extensive use of RWD in NICE guidance (15). NICE anticipates that RWD can reduce some of the uncertainties arising from limitations of randomised controlled trial (RCT) evidence, such as uncertain generalisability, the need to make indirect comparisons and limited long-term follow-up. There is a common ground that RWD can provide useful information in challenging circumstances, such as with small patient populations, rare diseases and cases where robust evidence is lacking, including single-arm trials (5,16).

Although the use of RWD in HTA appears to be relatively new, the use of RWD is not entirely new in NICE. RWD have already been used in diverse ways in NICE appraisals when developing their guidance. Registry or hospital data, commonly regarded as RWD, have been used for economic modelling. For example, NICE issued technology appraisal (TA) guidance for lenalidomide with rituximab for previously treated follicular lymphoma (NICE TA627) using UK registry data for the clinical outcomes of the comparators due to the absence of data from direct treatment comparison (17). Another example is European Chart Review data used to make indirect treatment comparisons when comparative data were absent in an appraisal of ibrutinib for treating Waldenstrom's macroglobulinaemia (NICE TA795) (18).

The use of RWD in NICE appraisals has been reviewed in several studies. A review of NICE guidance issued in 2015 and 2016, found limited use but increasing prominence of RWD (19). Also, RWD have been used to predict long-term effectiveness in the submissions to

For peer review only

different HTA agencies (20). Several studies have discussed the role of RWD in supporting healthcare decision-making and have broadly outlined the benefits and challenges of using RWD (21–23). However, these studies considered how RWD have been used in HTA decision-making based on case studies. One study reviewed the use of RWD in economic models in single technology appraisals (STAs) of cancer drugs more comprehensively (24). It found that RWD were extensively used in the cost-effectiveness analyses. However, the ability of this study to answer the question "How have RWD been used?" was limited since it only reported the number of times RWD were used as model inputs in appraisals.

To date, no study has shown how the use of RWD has changed over time or varied by type of cancer. Also, the extent to which RWD have been used to supplement the evidence in economic models has not been fully reviewed. This remains a gap in our understanding of the use of RWD. This study aims to provide a more complete picture of use of RWD in economic models for drug appraisals. The specific purposes of this study are to identify trends in the use of RWD over time and by type of cancer and to characterise patterns of use of RWD in economic models.

Methods

This study extracted data from 229 NICE STAs of oncology medicines for which NICE issued guidance between January 2011 and December 2021. The data were extracted following a protocol developed to document information about using RWD in economic models in NICE STAs of oncologic medicines (25). Extracted data include general information about STAs, evidence-specific information such as characteristics of primary clinical evidence, and the

For peer review only

For peer review only - http://bmjopen.bmj.com/site/about/guidelines.xhtml

use of RWD in the economic model. The definition of RWD in this study was data relating to patient health status and/or the delivery of health care routinely collected from non-experimental settings (25). The protocol extensively covers the components of economic models where RWD can be potentially used. While reporting the number of uses of RWD in each appraisal is a simple way to describe the use of RWD, this approach has limitations in understanding trends in the use of RWD in NICE STAs. A fuller picture of how RWD were used can be obtained by examining the pattern of use of RWD within appraisals. The data extraction protocol distinguishes 31 areas in economic evaluations where RWD can be used (Supplement 1). Use of RWD in these different areas was examined in order to look for common patterns.

Different uses of RWD and patterns are characterised by which components of an economic model were informed by RWD. There are three categories of use: any, non-parametric, and parametric use of RWD. Any use of RWD refers to its use in any part of the economic model regardless of how RWD were used. Parametric use means that RWD provides numerical values for specific variables in the economic model. For example, using data to estimate overall survival (OS) or resource use in the economic model is categorised as parametric use. Non-parametric use is where RWD are used to develop the economic model structure and to support assumptions in the model. Examples of non-parametric use include using RWD to select comparators or to validate the choice of survival distribution. This categorisation groups the uses and increases focus on where RWD were used in an economic model. Another advantage of this categorisation is that it facilitates comparison of the use of RWD across appraisals. This categorisation helps analyse where RWD were used systematically and compare the use between appraisals. This study investigated each category of use over

For peer review only - http://bmjopen.bmj.com/site/about/guidelines.xhtml

time and by type of cancer. Also, the association between number of non-parametric and number of parametric uses was examined using Spearman rank-order correlation.

Major and minor uses of RWD were identified based on which part of the economic evaluation they informed. Among 31 components included in the extraction protocol, three components (OS of intervention/comparators, volume of treatment of intervention/comparators, choice of comparators) were identified as major uses of RWD, which are highly likely to influence the estimated incremental cost-effectiveness ratio. This assumption was supported by experts' opinions (26). The remaining components were considered minor uses of RWD. This study highlights the sources of RWD used in these three major components to provide detailed information about the potential and limitations of using various sources of RWD. Common sources of RWD identified in the NICE RWE framework were used for this study: electronic health records, administrative data, registries, chart reviews and observational cohorts with primary data collection. Where the sources were not defined in the framework, they were classified in separate categories: namely data from the Office for National Statistics (ONS) and market share data. While the framework classifies health surveys, interviews and focus group as RWD, this study excluded them, following the definition of RWD, if the data are not routinely collected.

For peer review only

Results

The use of RWD in economic models

Figure 1 shows how use of RWD has changed 2011 to 2021. RWD were used in the economic models of most STAs. Parametric use of RWD was greater than non-parametric

For peer review only - http://bmjopen.bmj.com/site/about/guidelines.xhtml

use. Over the 11 years, an average of 76 % of appraisals made parametric use of RWD, whereas an average 41% of appraisals used RWD non-parametrically.

Figure 2 reports use of RWD by type of cancer. There was some difference between the non-parametric and parametric uses in the different types of cancer. In some appraisals, there was no non-parametric use of RWD, whereas for some types of cancer all appraisals made parametric use of RWD. These appraisals were found in the cancers with only a few STAs (thyroid cancer, genomic biomarker-based cancer, pancreatic cancer and neuroblastoma). Among cancer types where there were more than three STAs, the appraisals of skin cancer showed the most marked difference between the two uses. All the appraisals of skin cancer made parametric use of RWD. In comparison, non-parametric uses of RWD were made in only 27.3% of these appraisals.

The relationship between the two different uses was reviewed. Figure 3 shows the distribution of parametric and non-parametric uses of RWD per appraisal. In 43% of appraisals, parametric use was made when no non-parametric use was made. With respect to the association, no clear relationship is apparent in the heatmap. Spearman's rank correlation shows that non-parametric and parametric use are not statistically associated (r_s=0.07, p=0.28).

Patterns of use of RWD

Identified patterns of use of RWD, regardless of the type of use, are presented in Table 1. Patterns of use of RWD observed on a single occasion are not separately identified in the table but are grouped under *Others*. No dominant pattern of use of RWD in economic models was identified in these appraisals. Among identified patterns of using RWD (n=117),

For peer review only - http://bmjopen.bmj.com/site/about/guidelines.xhtml

only fifteen patterns appeared in more than two appraisals, cumulatively 51% of all appraisals. 16% of STAs did not use RWD in any part of the economic model. The second-most commonly observed pattern was the pattern estimating overall survival of intervention and comparators (6% of all patterns). The third-most common pattern was use of RWD to estimate end-of-life resource use (5% of all patterns).

Non-parametric and parametric use of RWD were separately reviewed (Supplement 2). Three-fifths of all appraisals made no non-parametric use of RWD. The commonest pattern of non-parametric use of RWD was to validate the choice of survival distribution for the intervention and comparators (9% of appraisals), followed by use of RWD to identify comparators (6% of appraisals). The patterns of parametric use of RWD were more diverse than the patterns of non-parametric use. 76% of appraisals used RWD to inform at least one parameter in the economic model. Using RWD for estimating end-of-life resource use was the commonest pattern (10% of appraisals), followed by the use of RWD to estimate OS for the intervention and comparators (7% of appraisals).

Sources of the real-world data

Figure 4 summarises the sources of RWD used for the major components of the economic evaluation. As sources of data are likely to be different for intervention and comparators in estimating OS and volume of treatment, the use of RWD was reviewed separately for the intervention and the comparators. Depending on the components, frequently used sources of RWD were different. Data from ONS were commonly used for estimating overall survival, whereas market share data were frequently a source of RWD for determining relevant comparators in economic models. These data were often collected by the company or private international health information entities such as IQVIA. Three sources of RWD

For peer review only

For peer review only - http://bmjopen.bmj.com/site/about/guidelines.xhtml

(registries, administrative data, chart reviews) were found across the three major components. On average, 22% of sources of RWD used for major components were registry data. The Surveillance, Epidemiology, and End Results Program (SEER) and Flatiron were frequently used registry data. Administrative data were the sources on 15% of the occasions that RWD were used for major components.

Clinician surveys or expert opinions are often used in economic models when determining the resource use or distribution of subsequent treatments. For example, the appraisal of nivolumab for previously treated unresectable advanced or recurrent oesophageal cancer (NICE TA707) used a clinician survey to determine the frequency of resource use in order to estimate disease management costs (27). However, data from clinician surveys or expert opinions are not classified as RWD following the definition used in this study.

Discussion

This study identified how RWD have been used in economic models in NICE appraisals of oncology medicines over time and by type of cancer, focusing on the specific ways that RWD have been used and patterns of RWD use. Most previous studies investigated the use of RWD in small samples of appraisals without explicit criteria for identifying RWD or tried to identify its use from stakeholder interviews. This study extracted explicit and systematic information on the use of RWD and assessed the use of RWD in economic models in NICE STAs of oncology medicines.

When looking at the use of RWD over time, it is notable that RWD have been used at least since 2011. NICE has accepted relevant data to assess new technologies. Regardless of data

For peer review only - http://bmjopen.bmj.com/site/about/guidelines.xhtml
For peer review only

types, it is standard practice to use the best available evidence in NICE technology appraisals (28). The key aim of NICE appraisals is to provide a summary of the relevant evidence to ensure that the appraisal committee can make fully informed decisions about the new technology (29). If RCTs offer limited information for decision-making, the evaluation may be strengthened by using alternative data, such as observational data. For this, it is important to use such data correctly. There is a NICE decision support unit (DSU) report and a technical support document to assist better analysis and interpretation of treatment effects from non-RCT studies (16,30). These documents guide the analysis of non-RCT data and observational data more generally to avoid potential biases, such as selection bias in the estimation of treatment effects. This highlights NICE's long-standing interest in using diverse sources of evidence for drug appraisals.

Having documented where RWD have been used, the uses were classified by what was informed by RWD. This study found that although RWD were widely used across the economic models they were less often used non-parametrically. This result can be reviewed with the findings about the sources of RWD in the three major components. Among identified diverse sources of RWD, registries and administrative data were used in a minority of appraisals. It might be challenging to identify evidence matching the population specified in the decision problem. Clinical experts' opinions were often used to justify assumptions regarding the non-parametric variables. Expert judgement, including expert opinions or expert elicitation can be helpful when relevant data are unlikely to exist, such as for rare cancers. However, inconsistent application and insufficient reporting of the process are concerns (31,32).

For peer review only - http://bmjopen.bmj.com/site/about/guidelines.xhtml

Along with expert opinions, RWD available in the NHS could provide more systematic information to describe routine practice better. An example is the Cancer Drugs Fund review appraisal of pembrolizumab (NICE TA766) (33). In the original appraisal, the subsequent treatment was one of the sources of uncertainty. The data on subsequent treatments from clinical trials were incomplete and did not reflect UK clinical practice. In the review appraisal, systemic anti-cancer therapy dataset was used to identify subsequent treatment after adjuvant pembrolizumab treatment, and clinicians supported the findings of the data. The committee concluded that the data, although immature, reflected practice. This example shows that RWD and expert opinion can provide more robust evidence to support decision-making.

The correlation between the use of RWD for parametric and non-parametric purposes was investigated. Interestingly, no significant correlation was found. It was expected that manufacturers might be willing to use RWD for non-parametric purposes when some parameters are estimated based on RWD. Similarly, fewer non-parametric uses are expected if manufacturers do not need to use RWD as a source of parameter values. However, the statistical results and the heatmap suggested no significant association between uses. This result may imply that the different sources of RWD differ in their ability to address uncertainties in the economic model. RWD used for non-parametric purposes might not contain enough information to inform parameters in the economic model. For example, market share data can help identify comparators by showing which treatment is frequently used, whereas such data cannot indicate the volume of treatment due to the missing information about treatment sequences. Moreover, the evidence in the manufacturer’s submission is often identified through a literature review. The identified

For peer review only

literature can provide some information required for the model in the manufacturer's submission. However, it may not be sufficient to provide all the information required in different part of economic models since literature only reports its outcome to answer its own research questions. If manufacturers more often directly explore RWD databases when developing their models, the association between parametric and non-parametric use will increase.

In this study, the most common pattern was no use of RWD and there was no dominant pattern among patterns involving at least one use of RWD. This suggests that there is no agreed approach to using RWD in STAs. Each appraisal raises different decision-making issues. Also, the ability of RWD to address a particular decision problem varies by appraisal context. Consequently, approaches and opportunities to incorporate supplementary data such as RWD will vary across appraisals. Although there was no dominant pattern of non-parametric use, about half of the identified patterns included uses of RWD to validate or corroborate the survival distributions for either the intervention or the comparators. This may be a direct consequence of the NICE DSU document issued in 2011 (last updated in 2013) that proposed that the model used to extrapolate survival could be justified using external data sources (34). Using RWD to validate or corroborate the survival distributions can be viewed as an attempt to validate the clinical plausibility of survival models using external data.

This study has focussed on the use of RWD in the base case modelling of cost-effectiveness. However, data were also extracted on the use of RWD in sensitivity analyses and the results are presented elsewhere (26). The use of RWD as supplementary data in sensitivity analyses was expected to be more common than in the base case since the remaining uncertainty

For peer review only

For peer review only - http://bmjopen.bmj.com/site/about/guidelines.xhtml

around parameters in an economic model is usually explored in sensitivity analyses using alternative evidence (35). However, parametric use of RWD in the sensitivity analysis was found in only a few appraisals. The interviews, conducted for the separate study (26), helps draw an implication. The manufacturer is less likely to present the RWD analysis results in the sensitivity analysis if there is no clear evidence that additional RWD are favourable to its outcome. Processing RWD can require significant resources in terms of collection and analysis. Suppose there is no incentive to use RWD in a sensitivity analysis. In that case, manufacturers prefer to use other published RCTs to explore the uncertainty in their model inputs and the survival distribution. There are a few cases where companies submit additional RWD in response to technical engagement or when forced to with a negative appraisal consultation document. However, this brings other problems as the quality of the data can be very poor, and reporting is not transparent.

While RWD could provide information reflecting routine clinical practice, the reliability of the RWD can be questioned in some cases. RWD are expected to provide information reflecting routine practice. However, depending on the timing and context of data collection, RWD may not accurately reflect routine practice. Due to changes in clinical practice or the small number of patients recruited, the RWD sample is potentially different from the whole population. In TA660, darolutamide with androgen deprivation therapy for treating hormone-relapsed non-metastatic prostate cancer (36), RWD were used to estimate healthcare resource by the target population. The external evidence review group was concerned that the study population was recruited over a wide time interval, which may have seen substantial changes in clinical practice, and that the sample was too small to accurately identify the clinical benefits since the primary outcome was obtained from 44

For peer review only - http://bmjopen.bmj.com/site/about/guidelines.xhtml

patients diagnosed with the specific indication. This example highlights that RWD do not always reflect current practice. How and what information was collected to answer the question is critical to the use of RWD in STAs.

This study has some limitations. The main one is that the information about the use of RWD was extracted from the manufacturer's submission. The extracted data did not necessarily reflect the committee’s preferences regarding sources of evidence. The data in the manufacturers’ submissions are primary evidence in the STA process. However, the Appraisal Committee do not necessarily accept the data presented by manufacturers. Their preferences regarding RWD could be different from those of the manufacturer. A study reviewing the Appraisal Committee’s preferences regarding RWD is in progress and may provide a more comprehensive understanding of the previous use of RWD in NICE STAs. Despite this limitation, this study contributes to understanding the use of RWD in appraisals. It is the first study to provide systematic information about use of RWD in economic modelling by reviewing appraisals of oncology drugs over 11 years.

RWD data can and have been used in the economic models, but to date there is no consistent or systematic way of using RWD. While the use of RWD in designing and validating economic models and helping to reduce uncertainty has been emphasised (15), this study found that non-parametric uses were less common than parametric uses and there were no dominant patterns of using such data. The NICE RWE framework can play a key role, such as encouraging more systematic use RWD and identifying when RWD can reduce uncertainties in economic model for drug appraisals. Despite the potential benefits of this framework, the extent to which it will facilitate the use of RWD to reduce uncertainty is unclear. A recent study found that RWD collected while cancer drugs were provided

For peer review only

through a managed access scheme in England had done little to reduce uncertainty (37). Further in-depth study on use of RWD in NICE appraisals could inform a discussion of the opportunities and limitations of the NICE RWE framework.

Conclusion

NICE has had a long-standing interest in the use of RWD in STAs. A systematic review of oncology appraisals suggests that RWD have been widely used in diverse parts of the economic models. Between 2011 and 2021, parametric use was more commonly found in economic models than non-parametric use. Nonetheless, there was no clear pattern in the way these data were used. As each appraisal involves a different decision problem and the ability of RWD to provide the information required for the economic modelling varies, appraisals will continue to differ with respect to their use of RWD.

The original protocol for the study

The protocol for this study is published; Kang J, Cairns J. Protocol for data extraction: how real-world data have been used in the National Institute for Health and Care Excellence appraisals of cancer therapy. BMJ Open. 2022;12:e055985. doi: 10.1136/bmjopen-2021-055985.

A funding statement

For peer review only

Jiyeon Kang is supported by the Centre for Cancer Biomarkers, University of Bergen, funded by the Research Council of Norway grant number (223250). The funder is not involved in any aspect of the study conduct or the decision to submit the paper for publication.

A competing interest statement

The authors declare no competing interest.

Authors contribution

Both authors contributed to conceptualising and designing the study. JK analysed the data and drafted the manuscript. JC revised the manuscript for important intellectual content and contributed to the methodology.

Data availability statement

The data analysed in this study are available on the National Institute for Health and Care Excellence website [https://www.nice.org.uk/guidance/published?ngt=Technology%20appraisal%20guidance&ndt=Guidance].

Ethics Statements

Patient consent for publication

For peer review only - http://bmjopen.bmj.com/site/about/guidelines.xhtml

Not applicable.

Ethics approval

This study was approved by the Ethics Committee of the London School of Hygiene and Tropical Medicine on 14 November 2019 (17315).

For peer review only

For peer review only - http://bmjopen.bmj.com/site/about/guidelines.xhtml

Reference

1. Singh G, Schulthess D, Hughes N, Vannieuwenhuyse B, Kalra D. Real world big data for clinical research and drug development. Vol. 23, Drug Discovery Today. Elsevier Ltd; 2018. p. 652–60.

2. NICE. NICE signs up to join the GetReal Institute | News and features [Internet]. NICE; Available from: https://www.nice.org.uk/news/article/nice-signs-up-to-join-the-getreal-institute

3. Batra A, Cheung WY. Role of real-world evidence in informing cancer care: lessons from colorectal cancer. Curr Oncol [Internet]. 2019;26(Suppl 1):S53. Available from: /pmc/articles/PMC6878934/

4. Mahendraratnam N, Mercon K, Gill M, Benzing L, McClellan MB. Understanding Use of Real-World Data and Real-World Evidence to Support Regulatory Decisions on Medical Product Effectiveness. Clin Pharmacol Ther [Internet]. 2022 Jan 1;111(1):150–4. Available from: https://pubmed.ncbi.nlm.nih.gov/33891318/

5. Hogervorst MA, Pontén J, Vreman RA, Mantel-Teeuwisse AK, Goettsch WG. Real World Data in Health Technology Assessment of Complex Health Technologies. Front Pharmacol [Internet]. 2022 Feb 10;13. Available from: /pmc/articles/PMC8866967/

6. Griffiths EA, Macaulay R, Vadlamudi NK, Uddin J, Samuels ER. The Role of Noncomparative Evidence in Health Technology Assessment Decisions. Value Health [Internet]. 2017 Dec 1;20(10):1245–51. Available from: https://pubmed.ncbi.nlm.nih.gov/29241883/

For peer review only

7. Makady A, Ham R ten, de Boer A, Hillege H, Klungel O, Goettsch W. Policies for Use of Real-World Data in Health Technology Assessment (HTA): A Comparative Study of Six HTA Agencies. Value Heal [Internet]. 2017 Apr 1;20(4):520–32. Available from: http://dx.doi.org/10.1016/j.jval.2016.12.003

8. Jao R, Jaksa A, Pontynen A, Wang X. Health Techonolgy Assessment (HTA) Agencies Consideration of Real World Evidence (RWE). Value Heal [Internet]. 2018 May 1;21:S7. Available from: http://www.valueinhealthjournal.com/article/S1098301518303322/fulltext

9. Orsini LS, Berger M, Crown W, Daniel G, Eichler HG, Goettsch W, et al. Improving Transparency to Build Trust in Real-World Secondary Data Studies for Hypothesis Testing—Why, What, and How: Recommendations and a Road Map from the Real-World Evidence Transparency Initiative. Value Heal [Internet]. 2020 Sep 1;23(9):1128–36. Available from: https://doi.org/10.1016/j.jval.2020.04.002

10. Garrison LP, Neumann PJ, Erickson P, Marshall D, Mullins CD. Using Real-World Data for Coverage and Payment Decisions: The ISPOR Real-World Data Task Force Report. Value Heal [Internet]. 2007 Sep 1;10(5):326–35. Available from: https://onlinelibrary.wiley.com/doi/full/10.1111/j.1524-4733.2007.00186.x

11. Caro JJ, Briggs AH, Siebert U, Kuntz KM. Modeling good research practices - Overview: A report of the ISPOR-SMDM modeling good research practices task force-1. Value Heal. 2012;15(6):796–803.

12. EUnetHTA. Publication of a Ja2 methodological guideline (2015). Internal Validity of Non-randomised Studies (NRS) on Interventions [Internet]. Available from:

For peer review only - http://bmjopen.bmj.com/site/about/guidelines.xhtml

https://www.eunethta.eu/publication-of-a-ja2-methodological-guideline-internal-validity-of-non-randomised-studies-nrs-on-interventions/

13. NICE. Increasing use of health and social care data in guidance development [Internet]. NICE; Available from: https://www.nice.org.uk/about/what-we-do/our-programmes/nice-guidance/nice-guidelines/how-we-develop-nice-guidelines/data-and-analytics-statement-of-intent

14. National Institute for Health and Care Excellence (NICE). NICE strategy 2021 to 2026: Dynamic, Collaborative, Excellent. 2021;

15. National Institute for Health and Care Excellence (NICE). NICE real-world evidence framework [Internet]. 2022. Available from: www.nice.org.uk/corporate/ecd9

16. Bell H, Wailoo AJ, Hernandez M, Grieve R, Faria R, Gibson L, et al. THE USE OF REAL WORLD DATA FOR THE ESTIMATION OF TREATMENT EFFECTS IN NICE DECISION MAKING REPORT BY THE DECISION SUPPORT UNIT [Internet]. 2016. Available from: www.nicedsu.org.uk

17. National Institute for Health and Care Excellence (NICE). TA627 Lenalidomide with rituximab for previously treated follicular lymphoma. NICE; 2020.

18. National Institute for Health and Care Excellence (NICE). TA795 Ibrutinib for treating Waldenstrom's macroglobulinaemia. NICE; 2022.

19. Glen F, Pulfer A. The Use of Real World Data for Nice Decision-Making: A Review of Submissions To Three Treatment Effectiveness Evaluation Programmes In 2015 And 2016. Value Heal. 2017 Oct 1;20(9):A693.

For peer review only - http://bmjopen.bmj.com/site/about/guidelines.xhtml

20. Makady A, van Veelen A, Jonsson P, Moseley O, D'Andon A, de Boer A, et al. Using Real-World Data in Health Technology Assessment (HTA) Practice: A Comparative Study of Five HTA Agencies. Pharmacoeconomics [Internet]. 2018 Mar 1;36(3):359–68. Available from: https://pubmed.ncbi.nlm.nih.gov/29214389/

21. Hampson G, Towse A, Dreitlein WB, Henshall C, Pearson SD. Real-world evidence for coverage decisions: Opportunities and challenges. J Comp Eff Res. 2018 Dec 1;7(12):1133–43.

22. Roberts MH, Ferguson GT. Real-World Evidence: Bridging Gaps in Evidence to Guide Payer Decisions. PharmacoEconomics - Open [Internet]. 2021 Mar 18;5(1):3–11. Available from: https://doi.org/10.1007/s41669-020-00221-y

23. Boyle JM, Hegarty G, Frampton C, Harvey-Jones E, Dodkins J, Beyer K, et al. Real-world outcomes associated with new cancer medicines approved by the Food and Drug Administration and European Medicines Agency: A retrospective cohort study. Eur J Cancer [Internet]. 2021 Sep 1;155:136–44. Available from: https://linkinghub.elsevier.com/retrieve/pii/S0959804921004226

24. Bullement A, Podkonjak T, Robinson MJ, Benson E, Selby R, Hatswell AJ, et al. Real-world evidence use in assessments of cancer drugs by NICE. Int J Technol Assess Health Care [Internet]. 2020 Aug 1;36(4):388–94. Available from: https://doi.org/10.1017/S0266462320000434

25. Kang J, Cairns J. Protocol for data extraction: how real-world data have been used in the National Institute for Health and Care Excellence appraisals of cancer therapy. BMJ Open [Internet]. 2022 Jan 1;12(1):e055985. Available from:

For peer review only

For peer review only - http://bmjopen.bmj.com/site/about/guidelines.xhtml

https://bmjopen.bmj.com/content/12/1/e055985

26. Kang J. Improving economic evaluation and decision-making for oncology drugs using real-world data. London School of Hygiene and Tropical Medicine; 2023.

27. National Institute for Health and Care Excellence (NICE). TA707 Nivolumab for previously treated unresectable advanced or recurrent oesophageal cancer . NICE; 2021.

28. George E. How real-world data compensate for scarce evidence in HTA. Z Evid Fortbild Qual Gesundhwes [Internet]. 2016;112:S23–6. Available from: https://pubmed.ncbi.nlm.nih.gov/27320024/

29. National Institute for Health and Care Excellence (NICE). NICE health technology evaluations: the manual [Internet]. NICE; Available from: https://www.nice.org.uk/process/pmg36/chapter/developing-the-guidance

30. Faria R, Alava MH, Manca A, Wailoo AJ, Uk WWNO. NICE DSU TECHNICAL SUPPORT DOCUMENT 17: THE USE OF OBSERVATIONAL DATA TO INFORM ESTIMATES OF TREATMENT EFFECTIVENESS IN TECHNOLOGY APPRAISAL: METHODS FOR COMPARATIVE INDIVIDUAL PATIENT DATA REPORT BY THE DECISION SUPPORT UNIT [Internet]. 2015. Available from: www.nicedsu.org.uk

31. Hunger T, Schnell-Inderst P, Sahakyan N, Siebert U. USING EXPERT OPINION IN HEALTH TECHNOLOGY ASSESSMENT: A GUIDELINE REVIEW. Int J Technol Assess Health Care [Internet]. 2016;32(3):131–9. Available from: https://pubmed.ncbi.nlm.nih.gov/27502308/

For peer review only

For peer review only - http://bmjopen.bmj.com/site/about/guidelines.xhtml

32. Grigore B, Peters J, Hyde C, Stein K. Methods to Elicit Probability Distributions from Experts: A Systematic Review of Reported Practice in Health Technology Assessment. PharmacoEconomics 2013 3111 [Internet]. 2013 Oct 9;31(11):991–1003. Available from: https://link.springer.com/article/10.1007/s40273-013-0092-z

33. National Institute for Health and Care Excllence. TA766 Pembrolizumab for adjuvant treatment of completely resected stage 3 melanoma. NICE; 2022.

34. Latimer N. NICE DSU TECHNICAL SUPPORT DOCUMENT 14: SURVIVAL ANALYSIS FOR ECONOMIC EVALUATIONS ALONGSIDE CLINICAL TRIALS-EXTRAPOLATION WITH PATIENT-LEVEL DATA REPORT BY THE DECISION SUPPORT UNIT. 2011; Available from: www.nicedsu.org.uk

35. Drummond MF, Sculpher MJ, Claxton K, Stoddart GL, Torrance GW. Methods for the Economic Evaluation of Health Care Programmes. 4th ed. Oxford: Oxford University Press; 2015.

36. National Institute for Health and Care Excellence (NICE). TA660 Darolutamide with androgen deprivation therapy for treating hormone-relapsed non-metastatic prostate cancer. NICE; 2020.

37. Kang J, Cairns J. “Don’t Think Twice, It’s All Right”: Using Additional Data to Reduce Uncertainty Regarding Oncologic Drugs Provided Through Managed Access Agreements in England. PharmacoEconomics - Open 2022 [Internet]. 2022 Sep 20;1–15. Available from: https://link.springer.com/article/10.1007/s41669-022-00369-9

For peer review only

For peer review only - http://bmjopen.bmj.com/site/about/guidelines.xhtml

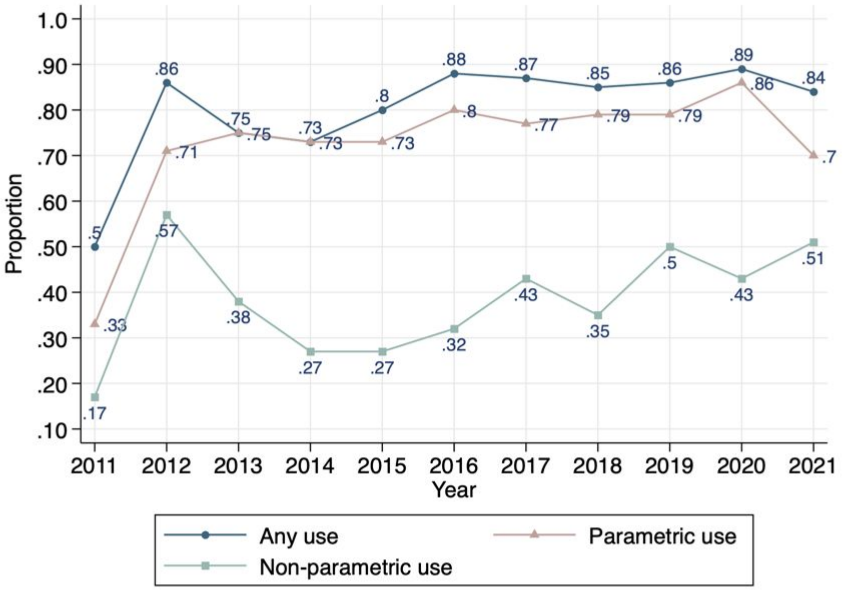

Figure 1 Parametric and non-parametric use of RWD over time

For peer review only - http://bmjopen.bmj.com/site/about/guidelines.xhtml

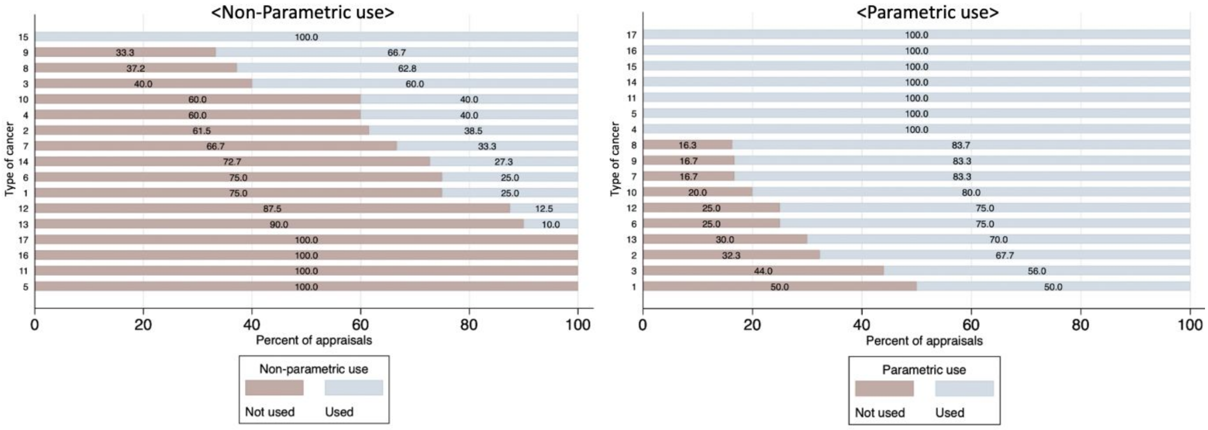

Type of cancer
1: Bladder cancer, 2: Blood & bone marrow cancer, 3: Breast cancer, 4: Colorectal cancer, 5: Neuroblastoma, 6: Head & Neck cancer, 7: Liver cancer, 8: Lung cancer, 9: Oesophageal cancer, 10: Ovarian cancer, 11: Pancreatic cancer, 12: Prostate cancer, 13: Renal cell carcinoma, 14: Skin cancer, 15: Stomach cancer, 16: Genomic biomarker-based cancer, 17: Thyroid cancer

Figure 2 Non-parametric and parametric use of RWD by type of cancer

For peer review only - http://bmjopen.bmj.com/site/about/guidelines.xhtml

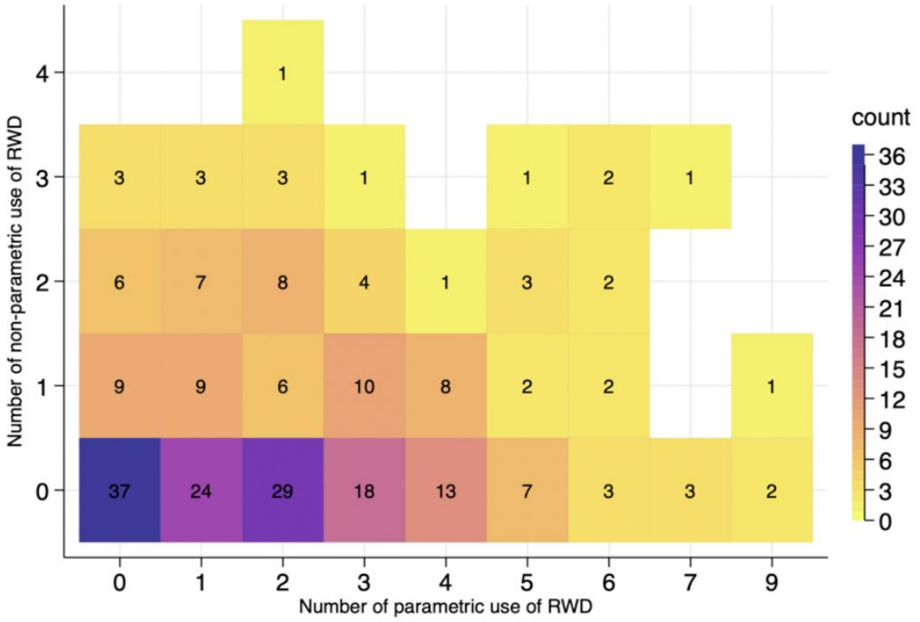

Figure 3 Parametric and non-parametric use of RWD by appraisal

peer review only

Table 1 Distribution of patterns of use of real-world data

Patterns	Number (%)
No use of RWD	37 (16.16%)
Estimating overall survival of intervention and comparators	13 (5.68%)
Estimating resource use of end-of-life	12 (5.24%)
Estimating resource use of end-of-life & health state cost	8 (3.49%)
Estimating resource use of health state cost	7 (3.06%)
Estimating overall survival of intervention and comparators and resource use of end-of-life & health state cost	6 (2.62%)
Estimating overall survival and progression-free survival of intervention and comparators and resource use of health state cost	5 (2.18%)
Validating survival distribution of intervention and comparators and estimating resource use of end-of-life	5 (2.18%)
Estimating overall survival and progression-free survival of intervention and comparators	5 (2.18%)
Estimating resource use of end-of-life and dose adjustment of intervention and comparators	4 (1.75%)
Estimating the volume of treatment of intervention and comparators	3 (1.31%)
Estimating overall survival of intervention and comparators and resource use of health state cost	3 (1.31%)
Validating survival distribution of intervention and comparators	3 (1.31%)
Choosing comparators	3 (1.31%)
Choosing comparators and estimating resource use of health state cost	3 (1.31%)
Others	112 (48.9%)
Total	229 (100%)

For peer review only - http://bmjopen.bmj.com/site/about/guidelines.xhtml

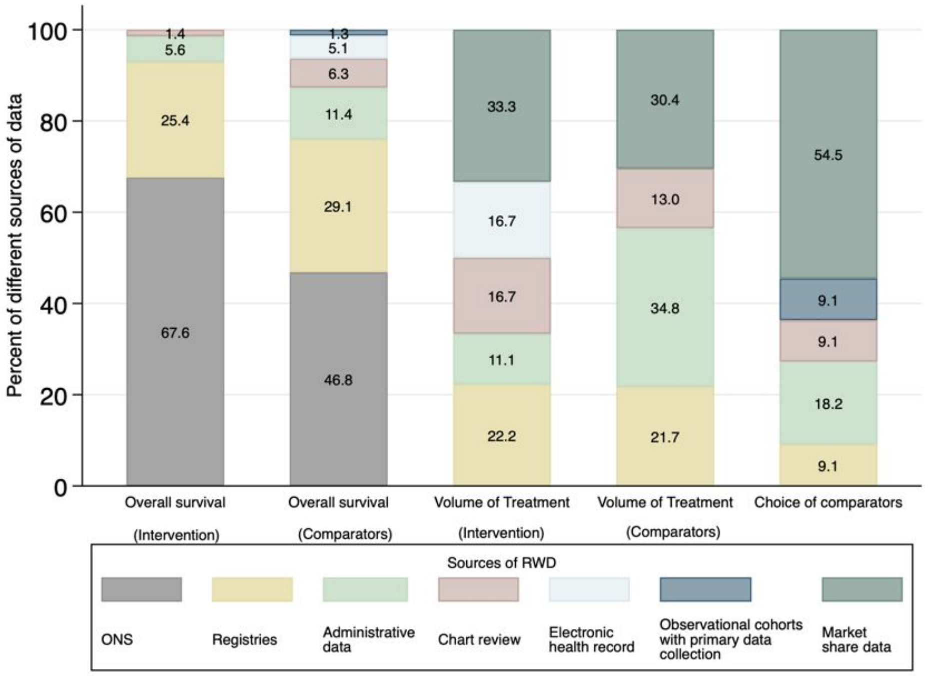

Figure 4 Sources of RWD used in three major components of the economic evaluation

For peer review only

Supplement 1. Components in use of real-world data in data extraction

Type of use	Elements
Non-parametric use	Characteristics of population
	Treatment sequence
	Choice of comparators
	Health state
	Model cycle
	Survival distribution (intervention)
	Survival distribution (comparators)
	Time-to-discontinuation (intervention)
	Time-to-discontinuation (comparators)
Parametric use	Overall survival (OS) of intervention
	Progression-free survival (PFS) of intervention
	Response rate (intervention)
	Time-to-progress (intervention)
	Adverse event (intervention)
	Overall survival (OS) of comparators
	Progression-free survival (PFS) of comparators
	Response rate (Comparators)
	Time-to-progress (Comparators)
	Adverse event (Comparators)
	Transition probability
	Health utility (generic measure)
	Health utility (cancer specific measure)
	Disutility
	Resource use of health state cost
	End-of-life resource use
	Resource use of adverse event cost (intervention)
	Volume of treatment (intervention)
	Dose adjustment (intervention)
	Resource use of adverse event cost (comparators)
	Volume of treatment (comparators)
	Dose adjustment (Comparators)

For peer review only

Supplement 2

Table 2A. Description of patterns of non-parametric use of real-world data

Pattern	Number (%)
No use of RWD	136 (59.39%)
Validating survival distribution of intervention and comparators	20 (8.73%)
Choice of comparators	14 (6.11%)
Validating survival distribution of comparators	13 (5.68%)
Treatment sequence	7 (3.06%)
Characteristics of population	7 (3.06%)
Validating survival distribution of intervention	4 (1.75%)
Treatment sequence & validating survival distribution of intervention and comparators	4 (1.75%)
Choice of comparator & validating survival distribution of intervention and comparators	3 (1.31%)
Choice of comparator & validating survival distribution of comparators & time-to-discontinuation of comparators	2 (0.87%)
Treatment sequence & time-to-discontinuation of intervention and comparators	2 (0.87%)
Treatment sequence & validating survival distribution of comparators	2 (0.87%)
Other*	15 (6.55%)
Total	229 (100%)

For peer review only

Supplement 2

Table 2B. Description of patterns of parametric use of real-world data

Patterns	Number (%)
No use of RWD	55 (24.02%)
Estimating end-of-life cost	23 (10.04%)
Estimating OS of intervention and comparators	17 (7.42%)
Using RWD for estimating end-of-life & health state cost	14 (6.11%)
Using RWD for estimating health state cost	13 (5.68%)
Estimating OS & PFS of intervention and comparators	10 (4.37%)
Estimating end-of-life cost & dose adjustment of intervention and comparators	9 (3.93%)
Using RWD for estimating OS of intervention and comparators & end-of-life & health state cost	6 (2.62%)
Estimating OS & PFS of intervention and comparators & health state cost	6 (2.62%)
Estimating volume of treatment of intervention and comparators	4 (1.75%)
Estimating OS & PFS of comparators	3 (1.31%)
Estimating OS of intervention and comparators & end-of-life cost	3 (1.31%)
Estimating OS of intervention and comparators & health-state cost	3 (1.31%)
Estimating OS & PFS of intervention and comparators & health state cost & end-of-life cost	3 (1.31%)
Other*	60 (26.2%)
Total	229 (100%)

For peer review only

For peer review only - http://bmjopen.bmj.com/site/about/guidelines.xhtml

BMJ Open

A cross-sectional analysis of use of real-world data in single technology appraisals of oncologic medicine by the National Institute for Health and Care Excellence in 2011 – 2021

Journal:	*BMJ Open*
Manuscript ID	bmjopen-2023-077297.R1
Article Type:	Original research
Date Submitted by the Author:	13-Dec-2023
Complete List of Authors:	Kang, Jiyeon; London School of Hygiene and Tropical Medicine Faculty of Public Health and Policy, Health Services Research and Policy<br>Cairns, John; London School of Hygiene and Tropical Medicine, Department of Health Services Research and Policy
**Primary Subject Heading**:	Health economics
Secondary Subject Heading:	Health economics, Health services research, Health policy
Keywords:	Health economics < HEALTH SERVICES ADMINISTRATION & MANAGEMENT, Health policy < HEALTH SERVICES ADMINISTRATION & MANAGEMENT, Decision Making, ONCOLOGY

SCHOLARONE™
Manuscripts

I, the Submitting Author has the right to grant and does grant on behalf of all authors of the Work (as defined in the below author licence), an exclusive licence and/or a non-exclusive licence for contributions from authors who are: i) UK Crown employees; ii) where BMJ has agreed a CC-BY licence shall apply, and/or iii) in accordance with the terms applicable for US Federal Government officers or employees acting as part of their official duties; on a worldwide, perpetual, irrevocable, royalty-free basis to BMJ Publishing Group Ltd ("BMJ") its licensees and where the relevant Journal is co-owned by BMJ to the co-owners of the Journal, to publish the Work in this journal and any other BMJ products and to exploit all rights, as set out in our licence.

The Submitting Author accepts and understands that any supply made under these terms is made by BMJ to the Submitting Author unless you are acting as an employee on behalf of your employer or a postgraduate student of an affiliated institution which is paying any applicable article publishing charge ("APC") for Open Access articles. Where the Submitting Author wishes to make the Work available on an Open Access basis (and intends to pay the relevant APC), the terms of reuse of such Open Access shall be governed by a Creative Commons licence – details of these licences and which Creative Commons licence will apply to this Work are set out in our licence referred to above.

Other than as permitted in any relevant BMJ Author's Self Archiving Policies, I confirm this Work has not been accepted for publication elsewhere, is not being considered for publication elsewhere and does not duplicate material already published. I confirm all authors consent to publication of this Work and authorise the granting of this licence.

For peer review only

A cross-sectional analysis of use of real-world data in single technology appraisals of oncologic medicine by the National Institute for Health and Care Excellence in 2011 – 2021

Jiyeon Kang[1], John Cairns[1]

[1]Department of Health Service Research and Policy,

Faculty of Public Health and Policy,

London School of Hygiene and Tropical Medicine, London, UK

Corresponding author: Jiyeon Kang (Jiyeon.Kang@lshtm.ac.uk)

15-17 Tavistock Place, London, WC1H 9SH, UK

Word count: 4,020

Keywords: Real-world data (RWD), Health Technology Assessment (HTA), single technology appraisal (STA), economic evaluation, oncology medicine, National Institute for Health and Care Excellence (NICE)

Abstract

Objectives This study aims to identify how real-world data (RWD) have been used in single-technology appraisals (STAs) of cancer drugs by the National Institute for Health and Care Excellence (NICE).

For peer review only

Design Cross-sectional study of NICE technology appraisals of cancer drugs for which guidance was issued between January 2011 and December 2021 (n=229). The appraisals were reviewed following a published protocol to extract the data about the use of RWD. The use of RWD was analysed by reviewing the specific ways in which RWD were used and by identifying different patterns of use.

Primary outcome measure The number of appraisals where RWD are used in the economic modelling.

Results Most appraisals used RWD in their economic models. The parametric use of RWD was commonly made in the economic models (76% of the included appraisals), whereas non-parametric use was less common (41%). Despite widespread use of RWD, there was no dominant pattern of use. Three sources of RWD (registries, administrative data, chart reviews) were found across the three important parts of the economic model (choice of comparators, overall survival and volume of treatment).

Conclusions NICE has had a long-standing interest in the use of RWD in STAs. A systematic review of oncology appraisals suggests that RWD have been widely used in diverse parts of the economic models. Between 2011 and 2021, parametric use was more commonly found in economic models than non-parametric use. Nonetheless, there was no clear pattern in the way these data were used. As each appraisal involves a different decision problem and the ability of RWD to provide the information required for the economic modelling varies, appraisals will continue to differ with respect to their use of RWD.

For peer review only

Strengths and limitations of this study

· This study investigated how real-world data (RWD) have been used in economic models of oncology medicines appraised by the National Institute for Health and Care Excellence based on 11 years of evidence.

· This study systematically applied a specially developed data extraction protocol.

· While the findings are likely to be less relevant to other disease areas, some insights with respect to the opportunities to use RWD and its limitations are transferrable.

Introduction

Real-world data (RWD) are increasingly being used in the life cycle of drug development (1). Clinical trials mainly generate the evidence to support decision-making in regulatory and health technology assessment (HTA), whereas RWD have been used complementarily for post-marketing surveillance. Interest in adopting and implementing RWD in the context of HTA has exploded in recent years (2) as the evidence gaps when relying heavily on clinical trials (3,4) are more widely recognised. Many HTA bodies view RWD as acceptable sources of data in the context of HTA (5,6). In most European HTA organisations, RWD are accepted to inform epidemiological data, cost, and resource use (7) and are used to fill evidence gaps by supporting the assumptions in economic models (8). Health economics and outcome research organisations, such as the European Network for HTA (EUnetHTA) and the International Society for the Professional Society for Health Economics and Outcomes Research (ISPOR), have been active in proposing how RWD can be collected and analysed in the HTA context (9–12).

For peer review only - http://bmjopen.bmj.com/site/about/guidelines.xhtml

The National Institute for Health and Care Excellence (NICE) has also shown increasing interest in RWD (13). NICE has highlighted the value of RWD and wants to use RWD to improve understanding of health care, resolve gaps in knowledge and drive early access to innovations (14). In June 2022, NICE introduced a real-world evidence (RWE) framework for the more extensive use of RWD in producing NICE guidance (15). NICE anticipates that RWD can reduce some of the uncertainties arising from limitations of randomised controlled trial (RCT) evidence, such as uncertain generalisability, the need to make indirect comparisons and limited long-term follow-up. There is a common ground that RWD can provide useful information in challenging circumstances, such as with small patient populations, rare diseases and cases where robust evidence is lacking, including single-arm trials (5,16).

Although the use of RWD in HTA appears to be relatively new, the use of RWD is not entirely new in NICE. RWD have already been used in diverse ways in NICE appraisals when developing their guidance. For example, NICE issued technology appraisal (TA) guidance for rituximab for follicular non-Hodgkin's lymphoma (TA226). In this TA, Government Actuary's Department life tables, based on the mortality experience of a population, were used for age and gender adjusted mortality. More recently, registry or hospital data, commonly regarded as RWD, have been used for economic modelling. TA guidance for lenalidomide with rituximab for previously treated follicular lymphoma (NICE TA627) using UK registry data for the clinical outcomes of the comparators due to the absence of data from direct treatment comparison (17). Another example is European Chart Review data used to make indirect treatment comparisons when comparative data were absent in an appraisal of ibrutinib for treating Waldenstrom's macroglobulinaemia (NICE TA795) (18).

For peer review only - http://bmjopen.bmj.com/site/about/guidelines.xhtml

The use of RWD in NICE appraisals has been reviewed in several studies. A review of NICE guidance published in 2015 and 2016, found limited use but increasing prominence of RWD (19). Also, RWD have been used to predict long-term effectiveness in the submissions to different HTA agencies (20). Several studies have discussed the role of RWD in supporting healthcare decision-making and have broadly outlined the benefits and challenges of using RWD (21–23). However, these studies considered how RWD have been used in HTA decision-making based on case studies. One study reviewed the use of RWD in economic models in single technology appraisals (STAs) of cancer drugs more comprehensively (24). It found that RWD were extensively used in the cost-effectiveness analyses. However, the ability of this study to answer the question "How have RWD been used?" was limited since it only reported the number of times RWD were used as model inputs in appraisals.

To date, no study has shown how the use of RWD has changed over time or varied by type of cancer. Also, the extent to which RWD have been used to supplement the evidence in economic models has not been fully reviewed. This remains a gap in our understanding of the use of RWD. This study aims to provide a more complete picture of use of RWD in economic models for cancer drug appraisals. The specific purposes of this study are to identify trends in the use of RWD over time and by type of cancer and to characterise patterns of use of RWD in economic models.

Methods

Data were extracted from 229 NICE STAs of oncology medicines for which NICE issued guidance between January 2011 and December 2021, following a protocol developed to

For peer review only - http://bmjopen.bmj.com/site/about/guidelines.xhtml

document information about using RWD in economic models in NICE STAs of oncologic medicines (25). Extracted data include general information about STAs, evidence-specific information such as characteristics of primary clinical evidence, and the use of RWD in the economic model. Information on the use of RWD was extracted for both the base-case and sensitivity analyses. However, only the use of RWD in the base-case is highlighted in this paper. The definition of RWD in this study was data relating to patient health status and/or the delivery of health care routinely collected from non-experimental settings (25). The protocol extensively covers the components of economic models where RWD can be potentially used. While reporting the number of uses of RWD in each appraisal is a simple way to describe the use of RWD, this approach has limitations in understanding trends in the use of RWD in NICE STAs. A fuller picture of how RWD have been used can be obtained by examining similarities and differences in use of RWD between appraisals. The pattern of use of RWD records which elements of the economic evaluation are informed by RWD. The data extraction protocol distinguishes 31 areas in economic modelling where RWD can be used (Supplement 1). These 31 areas are only considered as components for economic modelling in this paper. The use of RWD in these different areas in each appraisal was identified and reviewed for common patterns.

Different uses of RWD and patterns are characterised by which components of an economic model were informed by RWD. There are three categories of use: any, non-parametric, and parametric use of RWD. Any use of RWD refers to its use in any part of the economic model regardless of how RWD were used. Parametric use means that RWD provides numerical values for specific variables in the economic model. For example, using data to estimate overall survival (OS) or resource use in the economic model is categorised as parametric use.

For peer review only

Non-parametric use is where RWD are used to develop the economic model structure and to support assumptions in the model. Examples of non-parametric use include using RWD to select comparators or to validate the choice of survival distribution. Thus the label non-parametric implies that the RWD do not provide a parameter for the economic model, and is not referring to non-parametric methods. This categorisation groups the uses and increases focus on where RWD were used in the economic model, and facilitates comparison of the use of RWD across appraisals, for example, patterns of use over time and by type of cancer. Also, this categorisation enables examination of the association between the number of non-parametric and the number of parametric uses. When data are identified and used in synthesising evidence, the data can be used in multiple ways. Spearman rank-order correlation was carried out to test whether these two different ways of using RWD were associated.

Major and minor uses of RWD were identified based on which part of the economic evaluation they informed. Among 31 components included in the extraction protocol, three components (OS of intervention/comparators, volume of treatment of intervention/comparators, choice of comparators) were identified as major uses of RWD, which are highly likely to influence the estimated incremental cost-effectiveness ratio. This assumption was supported by experts' opinions (26). The remaining components were considered minor uses of RWD. This study highlights the sources of RWD used in these three major components to provide detailed information about the potential and limitations of using various sources of RWD. Common sources of RWD identified in the NICE RWE framework were used for this study: electronic health records, administrative data, registries, chart reviews and observational cohorts with primary data collection. Where the

For peer review only - http://bmjopen.bmj.com/site/about/guidelines.xhtml

sources were not defined in the framework, they were classified in separate categories: namely data from the Office for National Statistics (ONS) and market share data. While the framework classifies health surveys, interviews and focus group as RWD, this study excluded them, following the definition of RWD, if the data are not routinely collected.

Patient and Public Involvement

None of the patients or the public were involved in any part of this research.

Results

The use of RWD in economic models

Figure 1 shows how use of RWD has changed 2011 to 2021. RWD were used in the economic models of most STAs. Parametric use of RWD was greater than non-parametric use. Over the 11 years, an average of 76% of appraisals made parametric use of RWD, whereas an average 41% of appraisals used RWD non-parametrically.

Figure 2 reports use of RWD by type of cancer. There was some difference between the non-parametric and parametric uses in the different types of cancer. In some appraisals, there was no non-parametric use of RWD, whereas for some types of cancer all appraisals made parametric use of RWD. These appraisals were found in the cancers with only a few STAs (thyroid cancer, genomic biomarker-based cancer, pancreatic cancer and neuroblastoma). Among cancer types where there were more than three STAs, the appraisals of skin cancer showed the most marked difference between the two uses. All the

For peer review only - http://bmjopen.bmj.com/site/about/guidelines.xhtml

appraisals of skin cancer made parametric use of RWD. In comparison, non-parametric uses of RWD were made in only 27.3% of these appraisals.

The relationship between the two different uses was reviewed. Figure 3 shows the distribution of parametric and non-parametric uses of RWD per appraisal. In 43% of appraisals, parametric use was made when no non-parametric use was made. With respect to the association, no clear relationship is apparent in the heatmap. Spearman's rank correlation shows that non-parametric and parametric use are not statistically associated (r_s=0.07, p=0.28).

Patterns of use of RWD

Identified patterns of use of RWD, regardless of the type of use, are presented in Table 1. Patterns of use of RWD observed on a single occasion are not separately identified in the table but are grouped under *Others*. No dominant pattern of use of RWD in economic models was identified in these appraisals. Among identified patterns of using RWD (n=117), only fifteen patterns appeared in more than two appraisals, cumulatively 51% of all appraisals. 16% of STAs did not use RWD in any part of the economic model. The second-most commonly observed pattern was the pattern estimating overall survival of intervention and comparators (6% of all patterns). The third-most common pattern was use of RWD to estimate end-of-life resource use (5% of all patterns).

Table 1 Distribution of patterns of use of real-world data

Patterns	Number (%)
No use of RWD	37 (16.16%)

For peer review only - http://bmjopen.bmj.com/site/about/guidelines.xhtml

Estimating overall survival of intervention and comparators	13 (5.68%)
Estimating resource use of end-of-life	12 (5.24%)
Estimating resource use of end-of-life & health state cost	8 (3.49%)
Estimating resource use of health state cost	7 (3.06%)
Estimating overall survival of intervention and comparators and resource use of end-of-life & health state cost	6 (2.62%)
Estimating overall survival and progression-free survival of intervention and comparators and resource use of health state cost	5 (2.18%)
Validating survival distribution of intervention and comparators and estimating resource use of end-of-life	5 (2.18%)
Estimating overall survival and progression-free survival of intervention and comparators	5 (2.18%)
Estimating resource use of end-of-life and dose adjustment of intervention and comparators	4 (1.75%)
Estimating the volume of treatment of intervention and comparators	3 (1.31%)
Estimating overall survival of intervention and comparators and resource use of health state cost	3 (1.31%)
Validating survival distribution of intervention and comparators	3 (1.31%)
Choosing comparators	3 (1.31%)
Choosing comparators and estimating resource use of health state cost	3 (1.31%)
Others	112 (48.9%)
Total	229 (100%)

Non-parametric and parametric use of RWD were separately reviewed (Supplement 2). Three-fifths of all appraisals made no non-parametric use of RWD. The commonest pattern of non-parametric use of RWD was to validate the choice of survival distribution for the intervention and comparators (9% of appraisals), followed by use of RWD to identify comparators (6% of appraisals). The patterns of parametric use of RWD were more diverse than the patterns of non-parametric use. 76% of appraisals used RWD to inform at least one parameter in the economic model. Using RWD for estimating end-of-life resource use was

For peer review only - http://bmjopen.bmj.com/site/about/guidelines.xhtml

the commonest pattern (10% of appraisals), followed by the use of RWD to estimate OS for the intervention and comparators (7% of appraisals).

Sources of the real-world data

Figure 4 summarises the sources of RWD used for the major components of the economic evaluation. As sources of data are likely to be different for intervention and comparators in estimating OS and volume of treatment, the use of RWD was reviewed separately for the intervention and the comparators. Depending on the components, frequently used sources of RWD were different. Data from ONS were commonly used for estimating overall survival, whereas market share data were frequently a source of RWD for determining relevant comparators in economic models. These data were often collected by the company or private international health information entities such as IQVIA. Three sources of RWD (registries, administrative data, chart reviews) were found across the three major components. On average, 22% of sources of RWD used for major components were registry data. The Surveillance, Epidemiology, and End Results Program (SEER) and Flatiron were frequently used registry data. Administrative data were the source for 15% of the occasions that RWD were used for major components.

Clinician surveys or expert opinions are often used in economic models when determining the resource use or distribution of subsequent treatments. For example, the appraisal of nivolumab for previously treated unresectable advanced or recurrent oesophageal cancer (NICE TA707) used a clinician survey to determine the frequency of resource use in order to estimate disease management costs (27). However, data from clinician surveys or expert opinions are not classified as RWD following the definition used in this study.

For peer review only - http://bmjopen.bmj.com/site/about/guidelines.xhtml
For peer review only

Discussion

This study identified how RWD have been used in economic models in NICE appraisals of oncology medicines over time and by type of cancer, focusing on the specific ways that RWD have been used and patterns of RWD use. Most previous studies investigated the use of RWD in small samples of appraisals without explicit criteria for identifying RWD or tried to identify its use from stakeholder interviews. This study extracted explicit and systematic information on the use of RWD and assessed the use of RWD in economic models in NICE STAs of oncology medicines.

When looking at the use of RWD over time, it is notable that RWD have been used at least since 2011. Regardless of data types, it is standard practice to use the best available evidence in NICE technology appraisals (28). The key aim of NICE appraisals is to provide a summary of the relevant evidence to ensure that the appraisal committee can make fully informed decisions about the new technology (29). If RCTs offer limited information for decision-making, the evaluation may be strengthened by using alternative data, such as observational data. For this, it is important to use such data correctly. There is a NICE decision support unit (DSU) report and a technical support document to assist better analysis and interpretation of treatment effects from non-RCT studies (16,30). These documents guide the analysis of non-RCT data and observational data more generally to avoid potential biases, such as selection bias in the estimation of treatment effects. This highlights NICE's long-standing interest in using diverse sources of evidence for drug appraisals.

For peer review only

Having documented where RWD have been used, the uses were classified by what was informed by RWD. This study found that although RWD were widely used across the economic models they were less often used non-parametrically. This result can be reviewed with the findings about the sources of RWD in the three major components. Among identified diverse sources of RWD, registries and administrative data were used in a minority of appraisals. It might be challenging to identify evidence matching the population specified in the decision problem. Clinical experts' opinions were often used to justify assumptions regarding the non-parametric variables. Expert judgement, including expert opinions or expert elicitation can be helpful when relevant data are unlikely to exist, such as for rare cancers. However, inconsistent application and insufficient reporting of the process are concerns (31,32).

Along with expert opinions, RWD available in the NHS could provide more systematic information to describe routine practice better. An example is the Cancer Drugs Fund review appraisal of pembrolizumab (NICE TA766) (33). In the original appraisal, the subsequent treatment was one of the sources of uncertainty. The data on subsequent treatments from clinical trials were incomplete and did not reflect UK clinical practice. In the review appraisal, systemic anti-cancer therapy dataset was used to identify subsequent treatment after adjuvant pembrolizumab treatment, and clinicians supported the findings of the data. The appraisal committee concluded that the data, despite its immaturity, reflected practice. Although this cannot be generalised to all cases, for example, immature data with expert's opinions might reduce uncertainty regarding long-term survival, this example shows that in some cases, RWD and expert opinion can provide more robust evidence to support decision-making.

For peer review only - http://bmjopen.bmj.com/site/about/guidelines.xhtml

For peer review only

The correlation between the use of RWD for parametric and non-parametric purposes was investigated. Interestingly, no significant correlation was found. It was expected that manufacturers might be willing to use RWD for non-parametric purposes when some parameters are estimated based on RWD. Similarly, fewer non-parametric uses are expected if manufacturers do not need to use RWD as a source of parameter values. However, the statistical results and the heatmap suggested no significant association between uses. This result may imply that the different sources of RWD differ in their ability to address uncertainties in the economic model. RWD used for non-parametric purposes might not contain enough information to inform parameters in the economic model. For example, market share data can help identify comparators by showing which treatment is frequently used, whereas such data cannot indicate the volume of treatment due to the missing information about treatment sequences. Moreover, the evidence in the manufacturer's submission is often identified through a literature review. The identified literature can provide some information required for the model in the manufacturer's submission. However, it may not be sufficient to provide all the information required in different part of economic models since literature only reports its outcome to answer its own research questions. If manufacturers more often directly explore RWD databases when developing their models, the association between parametric and non-parametric use will increase.

In this study, the most common pattern was no use of RWD and there was no dominant pattern among patterns involving at least one use of RWD. This suggests that there is no agreed approach to using RWD in STAs. Each appraisal raises a different mix of decision-making issues. Also, the ability of RWD to address a particular decision problem varies by

For peer review only - http://bmjopen.bmj.com/site/about/guidelines.xhtml

appraisal context. Consequently, approaches and opportunities to incorporate supplementary data such as RWD will vary across appraisals. Although there was no dominant pattern of non-parametric use, about half of the identified patterns included uses of RWD to validate or corroborate the survival distributions for either the intervention or the comparators. This may be a direct consequence of the NICE DSU document issued in 2011 (last updated in 2013) that proposed that the model used to extrapolate survival could be justified using external data sources (34). Using RWD to validate or corroborate the survival distributions can be viewed as an attempt to validate the clinical plausibility of survival models using external data.

This study has focussed on the use of RWD in the base case modelling of cost-effectiveness. Data on the use of RWD in sensitivity analyses and the results are presented elsewhere (26). The use of RWD as supplementary data in sensitivity analyses was expected to be more common than in the base case since the remaining uncertainty around parameters in an economic model is usually explored in sensitivity analyses using alternative evidence (35). However, parametric use of RWD in the sensitivity analysis was found in only a few appraisals. The interviews, conducted for the separate study (26), helps draw an implication. The manufacturer is less likely to present the RWD analysis results in the sensitivity analysis if there is no clear evidence that additional RWD are favourable to its outcome. Processing RWD can require significant resources in terms of collection and analysis. Suppose there is no incentive to use RWD in a sensitivity analysis. In that case, manufacturers prefer to use other published RCTs to explore the uncertainty in their model inputs and the survival distribution. There are a few cases where companies submit additional RWD in response to technical engagement or when forced to with a negative appraisal consultation document.

For peer review only

However, this brings other problems as the quality of the data can be very poor, and reporting is not transparent.

While RWD could provide information reflecting routine clinical practice, the reliability of the RWD can be questioned in some cases. RWD are expected to provide information reflecting routine practice. However, depending on the timing and context of data collection, RWD may not accurately reflect routine practice. Due to changes in clinical practice or the small number of patients recruited, the RWD sample is potentially different from the whole population. In TA660, darolutamide with androgen deprivation therapy for treating hormone-relapsed non-metastatic prostate cancer (36), RWD were used to estimate healthcare resource by the target population. The external evidence review group was concerned that the study population was recruited over a wide time interval, which may have seen substantial changes in clinical practice, and that the sample was too small to accurately identify the clinical benefits since the primary outcome was obtained from 44 patients diagnosed with the specific indication. This example highlights that RWD do not always reflect current practice. How and what information was collected to answer the question is critical to the use of RWD in STAs.

This study has some limitations. The main one is that the information about the use of RWD was extracted from the manufacturer's submission. The extracted data did not necessarily reflect appraisal committee’s preferences regarding sources of evidence. The data in the manufacturers’ submissions are primary evidence in the STA process. However, appraisal committees do not necessarily accept the data presented by manufacturers. Their preferences regarding RWD could be different from those of the manufacturer. A study reviewing the appraisal committee’s preferences regarding RWD is in progress and may

For peer review only - http://bmjopen.bmj.com/site/about/guidelines.xhtml

provide a more comprehensive understanding of the previous use of RWD in NICE STAs. Despite this limitation, this study contributes to understanding the use of RWD in appraisals. It is the first study to provide systematic information about use of RWD in economic modelling by reviewing appraisals of oncology drugs over 11 years.

RWD data can and have been used in the economic models, but to date there is no consistent or systematic way of using RWD. While the use of RWD in designing and validating economic models and helping to reduce uncertainty has been emphasised (15), this study found that non-parametric uses were less common than parametric uses and there were no dominant patterns of using such data. The NICE RWE framework can play a key role, such as encouraging more systematic use of RWD and identifying when RWD can reduce uncertainties in economic model for drug appraisals. Despite the potential benefits of this framework, the extent to which it will facilitate the use of RWD to reduce uncertainty is unclear. A recent study found that RWD collected while cancer drugs were provided through a managed access scheme in England had done little to reduce uncertainty (37). Further in-depth study on use of RWD in NICE appraisals could inform a discussion of the opportunities and limitations of the NICE RWE framework.

Conclusion

NICE has had a long-standing interest in the use of RWD in STAs. A systematic review of oncology appraisals suggests that RWD have been widely used in diverse parts of the economic model. Between 2011 and 2021, parametric use was more commonly found in economic models than non-parametric use. Nonetheless, there was no clear pattern in the

For peer review only

way these data were used. As each appraisal involves a different decision problem and the ability of RWD to provide the information required for the economic modelling varies, appraisals will continue to differ with respect to their use of RWD.

The original protocol for the study

The protocol for this study is published; Kang J, Cairns J. Protocol for data extraction: how real-world data have been used in the National Institute for Health and Care Excellence appraisals of cancer therapy. BMJ Open. 2022;12:e055985. doi: 10.1136/bmjopen-2021-055985.

A funding statement

Jiyeon Kang is supported by the Centre for Cancer Biomarkers, University of Bergen, funded by the Research Council of Norway grant number (223250). The funder is not involved in any aspect of the study conduct or the decision to submit the paper for publication.

A competing interest statement

The authors declare no competing interest.

Authors contribution

For peer review only

Both authors contributed to conceptualising and designing the study. JK analysed the data and drafted the manuscript. JC revised the manuscript for important intellectual content and contributed to the methodology.

Data availability statement

The data analysed in this study are available on the National Institute for Health and Care Excellence website [https://www.nice.org.uk/guidance/published?ngt=Technology%20appraisal%20guidance&ndt=Guidance].

Ethics Statements

Patient consent for publication

Not applicable.

Ethics approval

This study was approved by the Ethics Committee of the London School of Hygiene and Tropical Medicine on 14 November 2019 (17315).

For peer review only

For peer review only - http://bmjopen.bmj.com/site/about/guidelines.xhtml

Reference

1. Singh G, Schulthess D, Hughes N, Vannieuwenhuyse B, Kalra D. Real world big data for clinical research and drug development. Vol. 23, Drug Discovery Today. Elsevier Ltd; 2018. p. 652–60.
2. NICE. NICE signs up to join the GetReal Institute | News and features [Internet]. NICE; Available from: https://www.nice.org.uk/news/article/nice-signs-up-to-join-the-getreal-institute
3. Batra A, Cheung WY. Role of real-world evidence in informing cancer care: lessons from colorectal cancer. Curr Oncol [Internet]. 2019;26(Suppl 1):S53. Available from: /pmc/articles/PMC6878934/
4. Mahendraratnam N, Mercon K, Gill M, Benzing L, McClellan MB. Understanding Use of Real-World Data and Real-World Evidence to Support Regulatory Decisions on Medical Product Effectiveness. Clin Pharmacol Ther [Internet]. 2022 Jan 1;111(1):150–4. Available from: https://pubmed.ncbi.nlm.nih.gov/33891318/
5. Hogervorst MA, Pontén J, Vreman RA, Mantel-Teeuwisse AK, Goettsch WG. Real World Data in Health Technology Assessment of Complex Health Technologies. Front Pharmacol [Internet]. 2022 Feb 10;13. Available from: /pmc/articles/PMC8866967/
6. Griffiths EA, Macaulay R, Vadlamudi NK, Uddin J, Samuels ER. The Role of Noncomparative Evidence in Health Technology Assessment Decisions. Value Health [Internet]. 2017 Dec 1;20(10):1245–51. Available from: https://pubmed.ncbi.nlm.nih.gov/29241883/

For peer review only

For peer review only - http://bmjopen.bmj.com/site/about/guidelines.xhtml

7. Makady A, Ham R ten, de Boer A, Hillege H, Klungel O, Goettsch W. Policies for Use of Real-World Data in Health Technology Assessment (HTA): A Comparative Study of Six HTA Agencies. Value Heal [Internet]. 2017 Apr 1;20(4):520–32. Available from: http://dx.doi.org/10.1016/j.jval.2016.12.003

8. Jao R, Jaksa A, Pontynen A, Wang X. Health Techonolgy Assessment (HTA) Agencies Consideration of Real World Evidence (RWE). Value Heal [Internet]. 2018 May 1;21:S7. Available from: http://www.valueinhealthjournal.com/article/S1098301518303322/fulltext

9. Orsini LS, Berger M, Crown W, Daniel G, Eichler HG, Goettsch W, et al. Improving Transparency to Build Trust in Real-World Secondary Data Studies for Hypothesis Testing—Why, What, and How: Recommendations and a Road Map from the Real-World Evidence Transparency Initiative. Value Heal [Internet]. 2020 Sep 1;23(9):1128–36. Available from: https://doi.org/10.1016/j.jval.2020.04.002

10. Garrison LP, Neumann PJ, Erickson P, Marshall D, Mullins CD. Using Real-World Data for Coverage and Payment Decisions: The ISPOR Real-World Data Task Force Report. Value Heal [Internet]. 2007 Sep 1;10(5):326–35. Available from: https://onlinelibrary.wiley.com/doi/full/10.1111/j.1524-4733.2007.00186.x

11. Caro JJ, Briggs AH, Siebert U, Kuntz KM. Modeling good research practices - Overview: A report of the ISPOR-SMDM modeling good research practices task force-1. Value Heal. 2012;15(6):796–803.

12. EUnetHTA. Publication of a Ja2 methodological guideline (2015). Internal Validity of Non-randomised Studies (NRS) on Interventions [Internet]. Available from:

For peer review only

For peer review only - http://bmjopen.bmj.com/site/about/guidelines.xhtml

https://www.eunethta.eu/publication-of-a-ja2-methodological-guideline-internal-validity-of-non-randomised-studies-nrs-on-interventions/

13. NICE. Increasing use of health and social care data in guidance development [Internet]. NICE; Available from: https://www.nice.org.uk/about/what-we-do/our-programmes/nice-guidance/nice-guidelines/how-we-develop-nice-guidelines/data-and-analytics-statement-of-intent

14. National Institute for Health and Care Excellence (NICE). NICE strategy 2021 to 2026: Dynamic, Collaborative, Excellent. 2021;

15. National Institute for Health and Care Excellence (NICE). NICE real-world evidence framework [Internet]. 2022. Available from: www.nice.org.uk/corporate/ecd9

16. Bell H, Wailoo AJ, Hernandez M, Grieve R, Faria R, Gibson L, et al. THE USE OF REAL WORLD DATA FOR THE ESTIMATION OF TREATMENT EFFECTS IN NICE DECISION MAKING REPORT BY THE DECISION SUPPORT UNIT [Internet]. 2016. Available from: www.nicedsu.org.uk

17. National Institute for Health and Care Excellence (NICE). TA627 Lenalidomide with rituximab for previously treated follicular lymphoma. NICE; 2020.

18. National Institute for Health and Care Excellence (NICE). TA795 Ibrutinib for treating Waldenstrom's macroglobulinaemia. NICE; 2022.

19. Glen F, Pulfer A. The Use of Real World Data for Nice Decision-Making: A Review of Submissions To Three Treatment Effectiveness Evaluation Programmes In 2015 And 2016. Value Heal. 2017 Oct 1;20(9):A693.

For peer review only - http://bmjopen.bmj.com/site/about/guidelines.xhtml

20. Makady A, van Veelen A, Jonsson P, Moseley O, D'Andon A, de Boer A, et al. Using Real-World Data in Health Technology Assessment (HTA) Practice: A Comparative Study of Five HTA Agencies. Pharmacoeconomics [Internet]. 2018 Mar 1;36(3):359–68. Available from: https://pubmed.ncbi.nlm.nih.gov/29214389/

21. Hampson G, Towse A, Dreitlein WB, Henshall C, Pearson SD. Real-world evidence for coverage decisions: Opportunities and challenges. J Comp Eff Res. 2018 Dec 1;7(12):1133–43.

22. Roberts MH, Ferguson GT. Real-World Evidence: Bridging Gaps in Evidence to Guide Payer Decisions. PharmacoEconomics - Open [Internet]. 2021 Mar 18;5(1):3–11. Available from: https://doi.org/10.1007/s41669-020-00221-y

23. Boyle JM, Hegarty G, Frampton C, Harvey-Jones E, Dodkins J, Beyer K, et al. Real-world outcomes associated with new cancer medicines approved by the Food and Drug Administration and European Medicines Agency: A retrospective cohort study. Eur J Cancer [Internet]. 2021 Sep 1;155:136–44. Available from: https://linkinghub.elsevier.com/retrieve/pii/S0959804921004226

24. Bullement A, Podkonjak T, Robinson MJ, Benson E, Selby R, Hatswell AJ, et al. Real-world evidence use in assessments of cancer drugs by NICE. Int J Technol Assess Health Care [Internet]. 2020 Aug 1;36(4):388–94. Available from: https://doi.org/10.1017/S0266462320000434

25. Kang J, Cairns J. Protocol for data extraction: how real-world data have been used in the National Institute for Health and Care Excellence appraisals of cancer therapy. BMJ Open [Internet]. 2022 Jan 1;12(1):e055985. Available from:

For peer review only

https://bmjopen.bmj.com/content/12/1/e055985

26. Kang J. Improving economic evaluation and decision-making for oncology drugs using real-world data. London School of Hygiene and Tropical Medicine; 2023.

27. National Institute for Health and Care Excellence (NICE). TA707 Nivolumab for previously treated unresectable advanced or recurrent oesophageal cancer . NICE; 2021.

28. George E. How real-world data compensate for scarce evidence in HTA. Z Evid Fortbild Qual Gesundhwes [Internet]. 2016;112:S23–6. Available from: https://pubmed.ncbi.nlm.nih.gov/27320024/

29. National Institute for Health and Care Excellence (NICE). NICE health technology evaluations: the manual [Internet]. NICE; Available from: https://www.nice.org.uk/process/pmg36/chapter/developing-the-guidance

30. Faria R, Alava MH, Manca A, Wailoo AJ, Uk WWNO. NICE DSU TECHNICAL SUPPORT DOCUMENT 17: THE USE OF OBSERVATIONAL DATA TO INFORM ESTIMATES OF TREATMENT EFFECTIVENESS IN TECHNOLOGY APPRAISAL: METHODS FOR COMPARATIVE INDIVIDUAL PATIENT DATA REPORT BY THE DECISION SUPPORT UNIT [Internet]. 2015. Available from: www.nicedsu.org.uk

31. Hunger T, Schnell-Inderst P, Sahakyan N, Siebert U. USING EXPERT OPINION IN HEALTH TECHNOLOGY ASSESSMENT: A GUIDELINE REVIEW. Int J Technol Assess Health Care [Internet]. 2016;32(3):131–9. Available from: https://pubmed.ncbi.nlm.nih.gov/27502308/

For peer review only

32. Grigore B, Peters J, Hyde C, Stein K. Methods to Elicit Probability Distributions from Experts: A Systematic Review of Reported Practice in Health Technology Assessment. PharmacoEconomics 2013 3111 [Internet]. 2013 Oct 9;31(11):991–1003. Available from: https://link.springer.com/article/10.1007/s40273-013-0092-z

33. National Institute for Health and Care Excllence. TA766 Pembrolizumab for adjuvant treatment of completely resected stage 3 melanoma. NICE; 2022.

34. Latimer N. NICE DSU TECHNICAL SUPPORT DOCUMENT 14: SURVIVAL ANALYSIS FOR ECONOMIC EVALUATIONS ALONGSIDE CLINICAL TRIALS-EXTRAPOLATION WITH PATIENT-LEVEL DATA REPORT BY THE DECISION SUPPORT UNIT. 2011; Available from: www.nicedsu.org.uk

35. Drummond MF, Sculpher MJ, Claxton K, Stoddart GL, Torrance GW. Methods for the Economic Evaluation of Health Care Programmes. 4th ed. Oxford: Oxford University Press; 2015.

36. National Institute for Health and Care Excellence (NICE). TA660 Darolutamide with androgen deprivation therapy for treating hormone-relapsed non-metastatic prostate cancer. NICE; 2020.

37. Kang J, Cairns J. "Don't Think Twice, It's All Right": Using Additional Data to Reduce Uncertainty Regarding Oncologic Drugs Provided Through Managed Access Agreements in England. PharmacoEconomics - Open 2022 [Internet]. 2022 Sep 20;1–15. Available from: https://link.springer.com/article/10.1007/s41669-022-00369-9

For peer review only

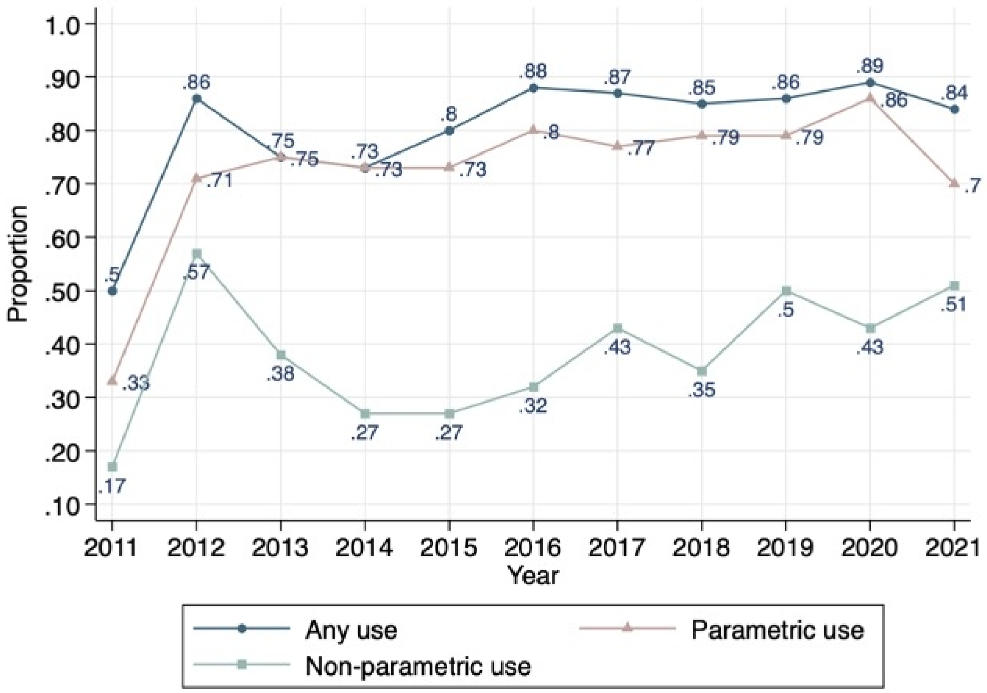

Parametric and non-parametric use of RWD over time

112x79mm (144 x 144 DPI)

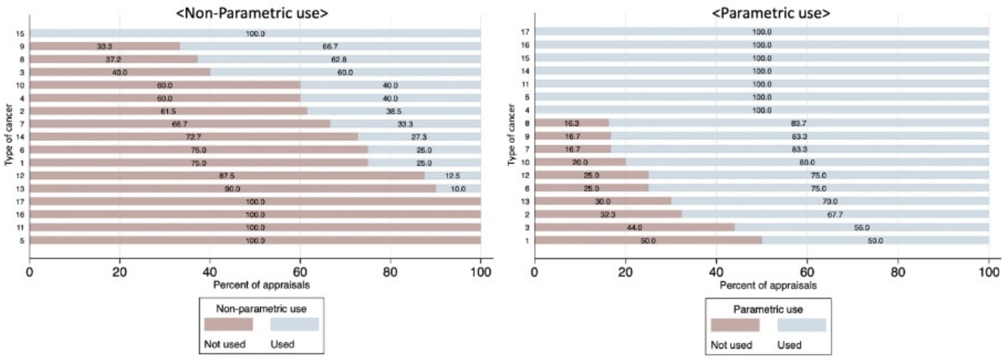

Type of cancer
1: Bladder cancer, 2: Blood & bone marrow cancer, 3: Breast cancer, 4: Colorectal cancer, 5: Neuroblastoma, 6: Head & Neck cancer, 7: Liver cancer, 8: Lung cancer, 9: Oesophageal cancer, 10: Ovarian cancer, 11: Pancreatic cancer, 12: Prostate cancer, 13: Renal cell carcinoma, 14: Skin cancer, 15: Stomach cancer, 16: Genomic biomarker-based cancer, 17: Thyroid cancer

Non-parametric and parametric use of RWD by type of cancer

159x70mm (144 x 144 DPI)

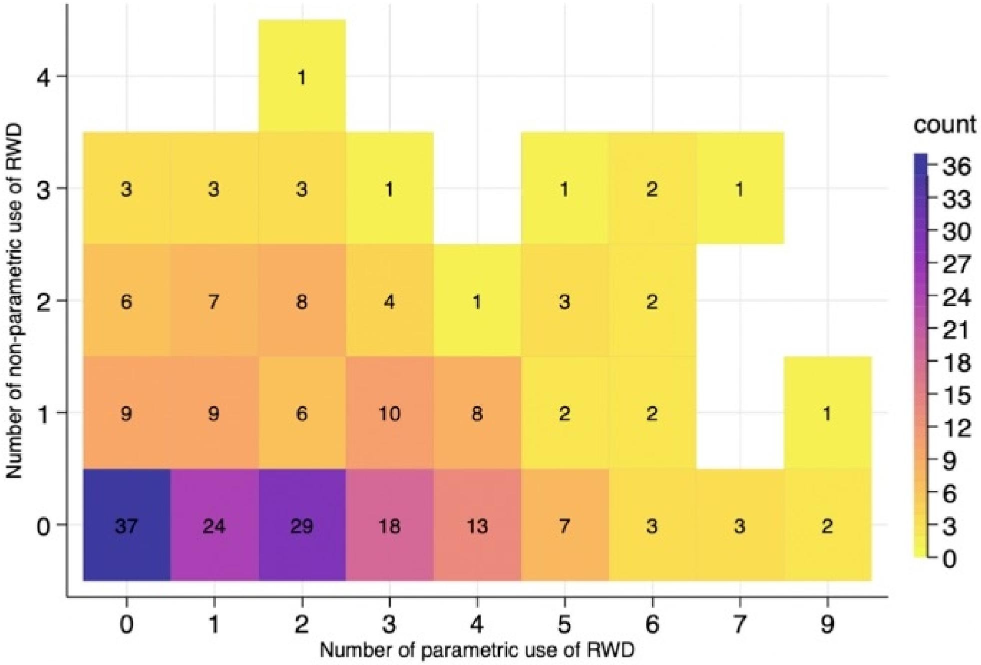

Parametric and non-parametric use of RWD by appraisal

123x85mm (144 x 144 DPI)

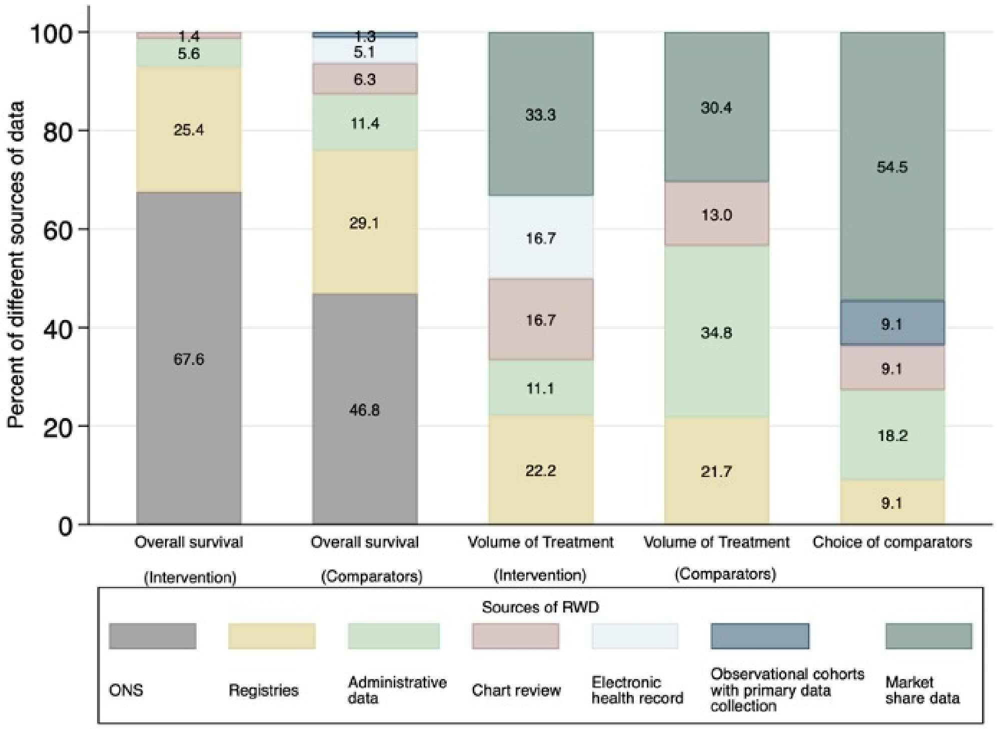

Sources of RWD used in three major components of the economic evaluation

122x88mm (144 x 144 DPI)

Supplement 1. Components in use of real-world data in data extraction

Type of use	Elements
Non-parametric use	Characteristics of population
	Treatment sequence
	Choice of comparators
	Health state
	Model cycle
	Survival distribution (intervention)
	Survival distribution (comparators)
	Time-to-discontinuation (intervention)
	Time-to-discontinuation (comparators)
Parametric use	Overall survival (OS) of intervention
	Progression-free survival (PFS) of intervention
	Response rate (intervention)
	Time-to-progress (intervention)
	Adverse event (intervention)
	Overall survival (OS) of comparators
	Progression-free survival (PFS) of comparators
	Response rate (Comparators)
	Time-to-progress (Comparators)
	Adverse event (Comparators)
	Transition probability
	Health utility (generic measure)
	Health utility (cancer specific measure)
	Disutility
	Resource use of health state cost
	End-of-life resource use
	Resource use of adverse event cost (intervention)
	Volume of treatment (intervention)
	Dose adjustment (intervention)
	Resource use of adverse event cost (comparators)
	Volume of treatment (comparators)
	Dose adjustment (Comparators)

For peer review only

Supplement 2

Table 2A. Description of patterns of non-parametric use of real-world data

Pattern	Number (%)
No use of RWD	136 (59.39%)
Validating survival distribution of intervention and comparators	20 (8.73%)
Choice of comparators	14 (6.11%)
Validating survival distribution of comparators	13 (5.68%)
Treatment sequence	7 (3.06%)
Characteristics of population	7 (3.06%)
Validating survival distribution of intervention	4 (1.75%)
Treatment sequence & validating survival distribution of intervention and comparators	4 (1.75%)
Choice of comparator & validating survival distribution of intervention and comparators	3 (1.31%)
Choice of comparator & validating survival distribution of comparators & time-to-discontinuation of comparators	2 (0.87%)
Treatment sequence & time-to-discontinuation of intervention and comparators	2 (0.87%)
Treatment sequence & validating survival distribution of comparators	2 (0.87%)
Other*	15 (6.55%)
Total	229 (100%)

For peer review only

Supplement 2

Table 2B. Description of patterns of parametric use of real-world data

Patterns	Number (%)
No use of RWD	55 (24.02%)
Estimating end-of-life cost	23 (10.04%)
Estimating OS of intervention and comparators	17 (7.42%)
Using RWD for estimating end-of-life & health state cost	14 (6.11%)
Using RWD for estimating health state cost	13 (5.68%)
Estimating OS & PFS of intervention and comparators	10 (4.37%)
Estimating end-of-life cost & dose adjustment of intervention and comparators	9 (3.93%)
Using RWD for estimating OS of intervention and comparators & end-of-life & health state cost	6 (2.62%)
Estimating OS & PFS of intervention and comparators & health state cost	6 (2.62%)
Estimating volume of treatment of intervention and comparators	4 (1.75%)
Estimating OS & PFS of comparators	3 (1.31%)
Estimating OS of intervention and comparators & end-of-life cost	3 (1.31%)
Estimating OS of intervention and comparators & health-state cost	3 (1.31%)
Estimating OS & PFS of intervention and comparators & health state cost & end-of-life cost	3 (1.31%)
Other*	60 (26.2%)
Total	229 (100%)

For peer review only - http://bmjopen.bmj.com/site/about/guidelines.xhtml
